# Emotional adaptation during a crisis: decline in anxiety and depression after the initial weeks of COVID-19 in the United States

**DOI:** 10.1038/s41398-021-01552-y

**Published:** 2021-08-20

**Authors:** Anastasia Shuster, Madeline O’Brien, Yi Luo, Laura A. Berner, Ofer Perl, Matthew Heflin, Kaustubh Kulkarni, Dongil Chung, Soojung Na, Vincenzo G. Fiore, Xiaosi Gu

**Affiliations:** 1 Department of Psychiatry, Icahn School of Medicine at Mount Sinai, New York, NY USA; 2 Nash Family Department of Neuroscience, Icahn School of Medicine at Mount Sinai, New York, NY USA; 3 Fralin Biomedical Research Institute at VTC, Virginia Tech, Roanoke, VA USA; 4 Department of Biomedical Engineering, UNIST, Ulsan, South Korea

**Keywords:** Depression, Predictive markers

## Abstract

Crises such as the COVID-19 pandemic are known to exacerbate depression and anxiety, though their temporal trajectories remain under-investigated. The present study aims to investigate fluctuations in depression and anxiety using the COVID-19 pandemic as a model crisis. A total of 1512 adults living in the United States enrolled in this online study beginning April 2, 2020 and were assessed weekly for 10 weeks (until June 4, 2020). We measured depression and anxiety using the Zung Self-Rating Depression scale and State-Trait Anxiety Inventory (state subscale), respectively, along with demographic and COVID-related surveys. Linear mixed-effects models were used to examine factors contributing to longitudinal changes in depression and anxiety. We found that depression and anxiety levels were high in early April, but declined over time. Being female, younger age, lower-income, and previous psychiatric diagnosis correlated with higher overall levels of anxiety and depression; being married additionally correlated with lower overall levels of depression, but not anxiety. Importantly, worsening of COVID-related economic impact and increase in projected pandemic duration exacerbated both depression and anxiety over time. Finally, increasing levels of informedness correlated with decreasing levels of depression, while increased COVID-19 severity (i.e., 7-day change in cases) and social media use were positively associated with anxiety over time. These findings not only provide evidence for overall emotional adaptation during the initial weeks of the pandemic, but also provide insight into overlapping, yet distinct, factors contributing to depression and anxiety throughout the first wave of the pandemic.

## Introduction

In early 2020, the novel coronavirus disease (COVID-19) devastated the globe with catastrophic health and economic consequences. People faced rapidly rising numbers of cases and deaths, overwhelmed healthcare systems, enormous economic strain, and staggering unemployment rates. All the while, individuals were asked to adhere to social distancing guidelines to reduce the chances of viral transmission. Thus, amidst the obvious threat to people’s physical health, the pandemic posed a dangerous risk to mental health. Indeed, historical precedence for the mental health consequences of pandemics has been well documented in prior research. Increased suicide rates were observed over the course of the 1918 Influenza pandemic, which racked our social, economic, and medical spheres in ways similar to the COVID-19 pandemic [1]. Research into mental health during the COVID-19 pandemic indicates that the current crisis is no exception. Worsening mental health conditions in adults have already been reported in the United Kingdom [2], United States [3], and Hong Kong [4].

However, humans often demonstrate incredible emotional adaptability to new situations, even when faced with prolonged hardship [5]. This is considered a form of resilience, which is defined as “the ability to withstand setbacks, adapt positively, and bounce back from adversity” [6]. Recent research has begun to elucidate the individual-level demographic and behavioral determinants of resilience and emotional adaptation. Particularly, increased resilience against developing depression has been linked to higher social support, familial support [7], and education levels [8], while being female [9] or of low socioeconomic status [10] puts one at a higher risk of developing depression. These individual differences are upheld during crises: following a widespread economic downturn, female and low-income individuals were more likely to develop depressive and anxious symptoms [8]. Other studies have shown that behavioral factors contribute to resilience as well: during the COVID-19 pandemic, a cross-sectional study of an Irish sample showed that emotional wellbeing is positively associated with participation in outdoor activities, and negatively associated with excessive intake of COVID-related social media content [11]. Yet, it remains unclear whether individuals will demonstrate such emotional adaptability over time during a prolonged crisis such as the COVID-19 pandemic; and if so, what factors might contribute to such adaptation.

Here, we examined longitudinal changes in depression and anxiety during the initial weeks of the pandemic (between April 2 and June 4, 2020) in a community sample in the United States. A total of 1512 participants enrolled in an online study on April 2nd and completed questionnaires every week for a 10-week period. Questions spanned a wide range of topics including self-reported depression and anxiety, subjective feelings and beliefs about the pandemic, and demographic information such as age and socioeconomic status (see Supplementary Information for a complete list). Following data collection, two mixed-effects general linear models were conducted to elucidate the variables contributing to fluctuations in depression and anxiety over the 10-week period. These analyses constitute one of the first investigations into both the static demographic (e.g., age, male/female) and dynamic (e.g., economic impact of COVID) factors associated with mental health during COVID-19.

## Materials and methods

The present study was part of a large web-based longitudinal study examining mental health and decision-making during the first wave of COVID-19 in the United States.

### Participants

**Fig. 1:**
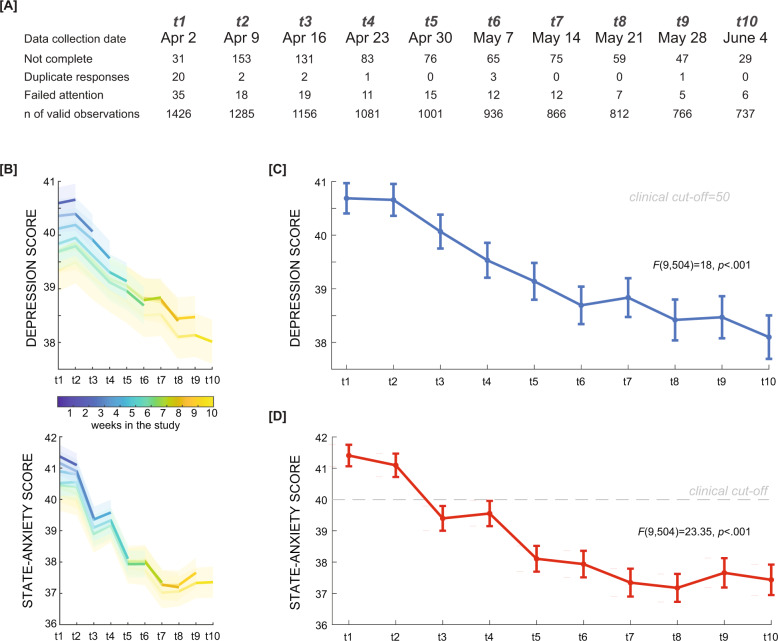
Data collection timeline, with participant exclusion, and depression and anxiety scores. Longitudinal data were collected through weekly surveys for a 10-week period. **A** Drop-outs are presented alongside numbers of exclusions based on duplicate responses and failed attention checks. **B** Depression and anxiety trends in different subsets of participants. The blue line depicts depression (top panel) and anxiety (bottom panel) scores from participants with valid observations from the first week of data collection (*n* = 1456). The yellow line depicts scores from participants who successfully completed all 10 weeks of data collection (*n* = 743). All other lines depict intermediate subsets of participants. The shaded area represents the standard error of the mean. **C** Depression and **D** state anxiety scores were measured weekly. Error bars indicate the standard error of the mean.

A total of 1512 participants who met eligibility criteria (age between 18–64, current US resident, >90% study participant approval rating) enrolled in the study on a web-based research platform (www.prolific.co) in a 24-h period beginning at 3 p.m. Eastern Time on April 2, 2020 (Fig. 1A). Exclusion criteria for observations were: (1) duplicated or corrupt entries (60 or 0.4% of observations), and (2) failure to respond accurately to an attention-check question embedded in the depression questionnaire (“If you are paying attention, please select ‘most of the time’”) (140 or .93% of observations). Valid data were obtained from a total of 1456 participants at the first time-point (716 females (49.18%), mean age 35.04 ± 13.08, from 50 US states and territories; see Table 1 for a summary of characteristics). The weekly dropout rate was between 3.90–11.72%. Dropped-out participants (*n* = 713) and final-sample participants (*n* = 743) did not differ with regard to demographic factors of sex, race, marital status, or income. The final sample was older (*t*(1, 454) = 6.1, *p* < 0.001), and reported lower depression and anxiety scores at the first time point (*t*(1 424) = 4.3, *p* < 0.001, *t*(1, 424) = 3.3, *p* < 0.001, respectively) (see Supplementary Table S1). There was also a smaller proportion of individuals with a lifetime anxiety disorder diagnosis in the dropout sample (*X*^2^(1, 456) = 4.7, *p* = 0.03). However, the trends of overall depression and anxiety scores of the full sample for each time point followed the trend of the subsample of completers (Fig. 1B).

**Table 1:** Sample demographic characteristics.

Sample demographic characteristics		
Characteristic	**No**.	**%**
Total, No.	1456	
Sex		
Male	740	50.8
Female/Other	716	49.2
Race		
White	1110	76.2
Non-White	346	23.8
Marriage status		
Married	493	33.8
Widowed/Divorced/Separated/Never married	963	66.2
Income (annual pre-COVID)		
<10k	98	6.7
10–20k	103	7.1
20–30k	137	9.4
30–40k	143	9.8
40–50k	126	8.6
50–60k	155	10.6
60–70k	98	6.7
70–80k	125	8.6
80–90k	77	5.3
90–100k	92	6.3
100–150k	204	14.0
>150k	98	6.7
Past or present mood disorder (major depressive disorder, bipolar disorder)		
Yes	308	21.2
No	1148	79.8
Past or present anxiety disorder (generalized anxiety disorder, panic disorder, social anxiety disorder, obsessive-compulsive disorder)		
Yes	342	23.5
No	1114	76.5
Lives alone		
Yes	158	10.9
No	1298	89.1
Has a child		
Yes	345	23.7
No	1111	76.3

Participants provided informed consent via an online form. The Institutional Review Board of the Icahn School of Medicine at Mount Sinai determined this research to be exempt following review. Participants received base compensation for their time each week ($7.25 for weeks that included behavioral task completion, $3 for weeks that included only survey completion), as well as a scaled bonus according to task performance. At week 5, participants received a $10 bonus for completing half of the study, and at week 10, participants received a $15 bonus for completing the entire study.

### Procedure

Each week, participants were allotted just over 2 h to complete questionnaires assessing mental health as well as perceptions of and behaviors related to the COVID-19 pandemic. Survey data collection was conducted within a 24-h time window every 7 days between April 2, 2020 and June 4, 2020 (ten time points in total). Participants additionally provided demographic information at the first time point. Other study elements included decision-making tasks, which are reported elsewhere as they are outside the scope of this study.

### Measures and scoring

The full survey, as seen by participants at the first time point, can be found in Supplementary Table S2. For the longitudinal analysis, we considered both static demographic factors (e.g., sex) and dynamic factors (fluctuating over time) as variables of interest in relation to mental health. The initial subset of demographic variables included sex, age, pre-COVID income level (binned into 12 discrete categories), a self-reported history of either a mood or an anxiety disorder diagnosis, and marital status. Age, sex, and race from our sample (*n* = 1456) were systematically examined in relation to population estimates from the 2019 US Census Bureau [12] (see Supplementary Table S3 for statistical details). In line with overall demographics of the web-based research platform used for the study, the current sample was significantly younger and more likely to be white than the median US population.

The Zung Self-Rating Depression scale [13] and State Anxiety Inventory [14] were used to assess depression and anxiety, respectively.

**Fig. 2:**
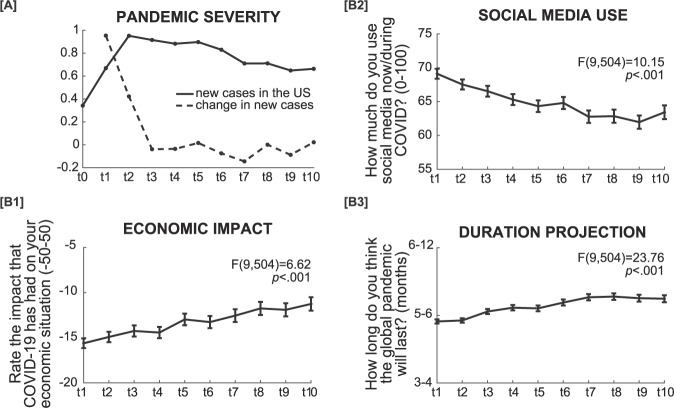
Dynamic variables in the study. **A** COVID-19 severity was measured as a 7-day running average of the number of daily new cases (per 10 K), and a weekly change in said average between timepoints. **B** Self-reported economic impact, social media frequency use, and subjective projection of pandemic’s duration during data collection. Statistics were calculated on participants with complete data (*n* = 743), but plots reflect every valid observation per time-point. The economic impact is overall negative but improves with time [B1]. Individuals reported using social media less frequently with time [B2]. Individuals’ projected duration for the pandemic increased with time [B3]. Error bars represent standard errors.

COVID-19 severity in the United States was computed as a 7-day running average of new daily cases [15]. We also calculated 7-day changes in COVID-19 cases by taking the national case count at time point *t* minus national case count at *t* − 1 (1 week earlier), divided by national case count at time point *t* − 1 (Fig. 2A).

Alongside demographic details, weekly answers to the following items were considered in the analysis (Fig. 2B1–3): (1) economic impact (“Rate the impact that COVID-19 has had on your economic situation”, rated from very negative impact, −50 to very positive impact, +50), (2) being informed (“How well are you keeping up with COVID-19 news?” rated from not at all informed, 0, to extremely well informed, +100), (3) social media use (“How much do you use social media now/during COVID?”, rated from not at all, 0, to all the time, +100), (4) and subjective projection of the pandemic’s duration (“How long do you think the global pandemic will last?”, 1–2 months, 3–4 months, 5–6 months, 6–12 months, or more than 1 year).

### Statistical analysis

Prior to analysis, we preprocessed the variables. We binarized sex into male and female (or other, *n* = 1), race into white and non-white, and marital status into married and not married. We created a mood disorder diagnosis variable by identifying participants with a past or present diagnosis of either major depression or bipolar disorder. We created an anxiety disorder diagnosis variable by identifying participants with a past or present diagnosis of either generalized anxiety disorder, panic disorder, social anxiety disorder, or obsessive-compulsive disorder. The economic impact was scaled to be between −0.5 and 0.5; informedness and social media use were scaled to 0–1.

To identify which variables were related to depression and anxiety, we conducted two linear mixed-effects models (see Supplementary Information and Supplementary Fig. S1 for reasons to choose these models based on model comparison): Depression (or anxiety) ~1 + age + sex + race + income + diagnosis + time + COVID-19 severity + economic impact + informedness + social media + COVID-19 future + (1 + time | participant). We then tested if the addition of time-by-demographic variable interactions improved model fit.

We also explored whether the addition of three other demographic variables (marital status, parental status, and living situation during the pandemic—alone or with other people) and pandemic policy variables (number of days under stay-at-home orders and since nonessential businesses were closed) improved model fit (see Supplementary Information and Supplementary Fig. S1).

All analyses were carried out using MATLAB 2018b [16], R 4.0.5 [17], and RStudio 1.4.1106 [18]. MATLAB was used for data handling, repeated measures analyses of variance, and plotting. R and RStudio were used for mixed-effect models. Mixed-effects models were conducted using the *lme4* and *lmerTest* packages in R [19, 20], with *p* values approximated via Satterthwaite’s degrees of freedom method. All linear mixed-effects were estimated using full information maximum likelihood (FIML) and included participants with partial data. Data were assumed missing at random conditional on covariates included in the model [21, 22]. Repeated-measures analyses of variance, which cannot accommodate missing data, were carried out using case-wise deletion of participants with any missing data or failed attention checks (*n* = 657).

We compared our main depression and anxiety models with various extended models using the *anova* function, implemented in R. We added variables of interest one by one to examine whether they helped explain the data (i.e., improved the model’s fit), compared against the main model. Each comparison resulted in chi-square and *p* values, reflecting the difference between the models’ fits.

## Results

Both depression and anxiety scores were highest at the beginning of the pandemic, and declined over the 10 weeks (repeated-measures ANOVA, *F*(9, 504) = 18.10, *p* < 0.001, partial *η*^2^ = 0.027 and *F*(9, 504) = 26.00, *p* < 0.001, partial *η*^2^ = 0.039, respectively; Fig. 1C, D). Notably, while average depression scores remained below the standard clinical cutoff for a depression diagnosis [23], the average anxiety score at the first time point (41.41 ± 13) exceeded a widely used clinical cutoff of 40, indicating that the average participant in our study was clinically anxious in early April 2020 (*t*(1, 425) = 4.1, *p* < 0.001, one sample *t*-test against 40) [24].

**Fig. 3:**
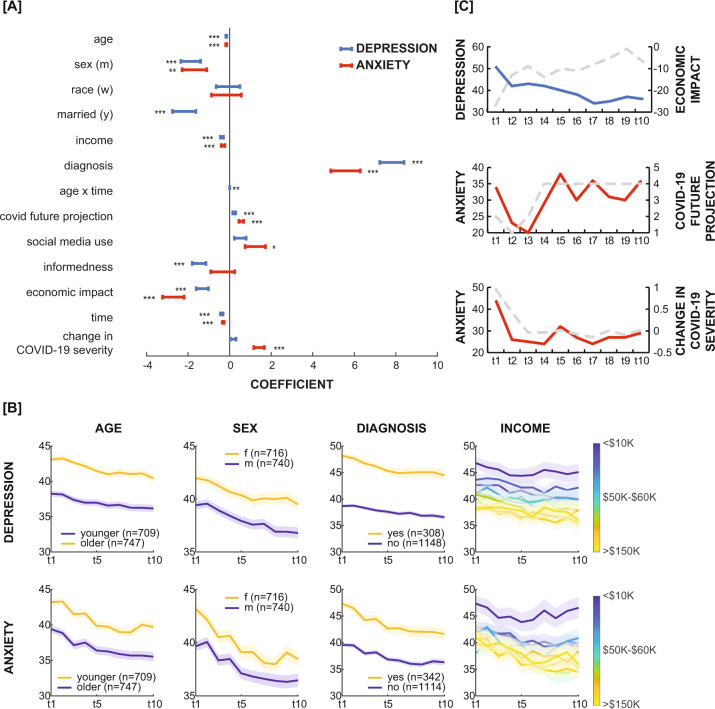
Factors influencing depression and anxiety during the COVID-19 pandemic in the United States. **A** The coefficients of a mixed-effects linear regression of depression (blue) and anxiety (red). Error bars represent confidence intervals. * *p* < 0.05, **p < 0.01, ***p < 0.001. **B** Illustration of significant demographic variables related to depression and anxiety. Numbers in parentheses represent the number of participants at the first time point, and plots are of all valid observations at each time point. **C** Example participants depicting significant behavioral and attitude variables related to depression and anxiety. Colored lines represent the example participant’s depression (blue) and anxiety (red). Dotted lines represent the same participant’s behavioral/attitude variable.

We used two linear mixed-effects models to estimate the influence of demographic and dynamic variables on depression and anxiety separately. We found similar demographic variables associated with depression and anxiety. Specifically, higher levels of depression and anxiety at each time point were related to being younger (β = −0.16, *t*(1, 478) = −7.8, *p* < 0.001; β = −0.16, *t*(1, 473.9) = −6.9, *p* < 0.001), female (β = −1.88, *t*(1, 451) = −3.9, *p* *<* 0.001; β = −1.69, *t*(1, 433.2) = −2.9, *p* *<* 0.001), and having lower income (β = −0.37, *t*(1, 447) = −5.1, *p* < 0.001; β = −0.32, *t*(1, 422.2) = −3.7, *p* < 0.001). As expected, having a past or present diagnosis of a mood or anxiety disorder was also related to a respective increase in depression (β = 7.81, *t*(1, 451) = 13.4, *p* < 0.001) or anxiety (β = 5.58, *t*(1, 443.2) = 7.9, *p* < 0.001). Finally, the addition of marital status to the model only improved the fit of the depression model, but not the anxiety model, and was therefore only included in the depression model. We found that being married was associated with reduced depression scores (β = −2.19, *t*(1, 451) = −3.9, *p* < 0.001), but not anxiety scores, over time (Fig. 3A).

For dynamic factors, we found both overlapping and distinct factors predicting changes in depression and anxiety, over and above the effect of time (Fig. 3A). The economic impact of COVID-19 negatively affected mental health, such that COVID-related worsening of one’s financial situation was related to increases in both depression (β = −1.31, *t*(9, 357) = −4.5, *p* < 0.001) and anxiety (β = −2.71, *t*(9, 849.3) = −5.2, *p* *<* 0.001). Likewise, changes in the subjective projection of the pandemic’s duration were positively associated with changes in depression and anxiety scores (β = 0.22, *t*(9, 664) = 3.4, *p* *=* 0.001 and β = 0.56, *t*(9, 248.5) = 5.0, *p* *<* 0.001, respectively). Interestingly, changes in anxiety, but not depression, were affected by social media use (β = 1.24, *t*(9, 962.2) = 2.5, *p* = 0.012), as well as the 7-day change in COVID-19 cases (β = 1.42, *t*(8, 334.9) = 5.6, *p* *<* 0.001), but not case count itself (Fig. 2A). Subjective levels of informedness were negatively associated with depression (β = −1.47, *t*(9, 171) = −4.5, *p* < 0.001), but not anxiety.

Next, we examined whether the interaction of static variables with time had any explanatory power over changes in mental health—in other words, whether the addition of interaction terms with time improved either model. Only the addition of the age-by-time interaction improved the fit of the depression model (*Χ*^2^: 9.55, *p* = .002). The age-by-time interaction was significantly positive (β = 0.004, *t*(878.3) = 3.1, *p* = 0.002), suggesting that although older individuals were less depressed overall, time had a less palliative effect on them as compared with younger individuals. No other time interactions could explain changes in depression, and none explained changes in anxiety in our models (see Supplementary Fig. S2 for coefficient values influencing depression and anxiety using the winning depression model variables).

## Discussion

Despite the known impact of crises and disasters on mental health, humans are able to adapt to hardship over time [5]. This study provides the first evidence that in the United States, depression and anxiety initially peaked but then declined over 10 weeks during the first wave of COVID-19. Furthermore, we report that overlapping, yet distinct, socioeconomic and psychological factors affected depression and anxiety trajectories as the pandemic lingered. Specifically, fluctuations in both depression and anxiety were associated with financial hardship and subjective projection of pandemic duration: while subjective projection might be related to mental health outcomes in a causal or consequential manner, the contribution of financial hardship to worsening mental health constitutes a potential causal effect of socioeconomic status on depression and anxiety. Changes in anxiety, but not depression, were associated with social media use and the 7-day change in national COVID-19 cases. Finally, changes in depression scores, but not anxiety scores, were influenced by personal informedness about the COVID-19 pandemic and did not decrease as much in older adults.

Consistent with past work [25, 26] on the link between economic recession and mental health, the worsening of the economic impact of COVID-19 on individuals was associated with increases in depression and anxiety scores in the current study. Following the 2008 Great Recession, a large body of research was conducted to investigate the proximal and long-term emotional ramifications of economic hardship. Financial insecurity and unemployment were persistently found to increase suicide prevalence [25, 27, 28] and decrease self-reported happiness and life satisfaction [29]. One investigation into suicide rates in Iceland following the 2008 recession did not find a significant uptick in suicides, a result that the authors attribute in part to “a strong welfare system and investing in social protection” [30]. Lower-income and financial insecurity were also associated with increased depression [24], and psychiatric hospitals reported an increase in outpatient counts among previously healthy individuals, as well as among those with anxiety, mood, and adjustment disorders [26]. In the context of COVID-19, complaints of increased self-reported depression and anxiety at the beginning of the pandemic were found to be associated with a lower societal appreciation for one’s occupation and the sudden onset of economic hardship [31]. Given the substantial—and potentially causal—relationship between economic crisis and mental health, especially with regard to income, the field would benefit from future investigations into potential mitigating effects of governmental financial support.

Several variables affected changes in anxiety but not depression, or vice versa, which could provide insight into the differences between depression and anxiety. Historically, scientists have vacillated between consideration of depression and anxiety as the same disorder, different disorders located along the same affective spectrum, and entirely different disorders with unique symptomatology [32, 33]. Depression and anxiety are very often comorbid in patients with psychiatric disorders [32], and until recently, self-report measures have rarely been able to distinguish between their symptoms [34]. But pharmacological separation exists in their treatments (antidepressants and anxiolytics, respectively) [33], and both overlapping and distinct neurobiological profiles have been found to be associated with depression and anxiety in neuroimaging studies [35]. Traditionally, depression has been associated with hopelessness and helplessness [35], whereas anxiety is often characterized by uncertainty and fear of the unknown [36, 37]. Accompanying a recent increased focus on the overlapping qualities of psychiatric conditions, theoretical frameworks of comorbid anxiety and depression hypothesize that they are both driven by beliefs about uncontrollability [36]. This prior work could, in part, illuminate why certain dynamic factors in the current study contributed to anxiety, depression, or both. For example, increased COVID-19 severity and social media use were singularly associated with exacerbated anxiety; this distinction might be explained in part by a vicious cycle of uncertainty that accompanied rising case numbers and nervous responses to the daily news by one’s peers on social media. On the other hand, levels of depression, but not anxiety, were lower among those who felt more informed about the pandemic, reflecting a relationship between obtaining information and infusing a sense of hopefulness. Finally, the dynamic variables that contributed to both anxiety and depression can potentially be associated with uncontrollability. Indeed, experiencing worsening financial hardship and ambiguity about the ability to support oneself in the future might instill feelings of uncertainty, and the pessimistic projection of a long-enduring pandemic timeline could make one believe that there is no end in sight. However, such claims are speculative without investigation into the specific differences between depression and anxiety that enabled certain factors to contribute to worsening scores for one disorder but not the other.

In accordance with recent research, we also found that age, sex, and income level were associated with overall depression and anxiety scores. Our results mirror those reported in other studies: a cohort of adults in the United Kingdom who had already completed a mental health study pre-COVID reported higher levels of mental distress during the pandemic, especially young and female participants, in addition to participants with small children [2]. However, while sex affected overall depression and anxiety scores, there was no significant interaction between sex and time, suggesting that females were more anxious and depressed overall but their rates of change in anxiety and depression over time were similar to males. This raises the possibility that female vulnerability to mental distress might not necessarily imply a disadvantage in their ability to bounce back (i.e., resilience). Differences in mental health by age have also been elucidated in prior work. Young adults in the United States were found to have high depression, anxiety, and posttraumatic stress disorder (PTSD) scores during the pandemic, with exacerbated loneliness and COVID-related anxiety increasing the likelihood that depression, anxiety, and PTSD scores would reach clinical threshold [38]. In another United States adult sample, depression symptoms were abnormally high at the beginning of the COVID-19 pandemic in comparison to pre-COVID national averages, and individuals with lower income levels reported more depression symptoms than their high-income counterparts [3]. Likewise, self-reported depression and health anxiety symptoms increased particularly rapidly in individuals who experienced financial insecurity in the early days of the pandemic [31]. Thus, the findings reported here provide evidence for individual differences in susceptibility to increased depression and anxiety during a crisis.

There are a number of limitations of the present study. First, no baseline scores for self-reported depression or anxiety could be established due to the unexpected nature of the COVID-19 pandemic and time needed to set up the study. To provide an approximation for national pre-pandemic levels of anxiety and depression, we compared scores from the current sample with previously-reported mean community scores of the same depression and anxiety measures that were published in articles unaffiliated with the present study. Anxiety and depression levels in our sample began at higher levels than previously reported community averages (though only significantly so for anxiety) and decreased to meet these averages by week 3 of data collection. The second limitation of our study is its correlational nature: with the exception of demographic information and the effects of time and 7-day change of COVID-19 cases, causation in either direction cannot be ascribed to the results of our mixed-effects models. For example, while our findings included an association between increased social media use and exacerbated anxiety scores, this could be explained either by social media content driving anxiety or by the likelihood that an anxious individual would monitor social media more closely. Likewise, the negative economic impact could contribute to depression, but increased depression could also render an individual unable to maintain their pre-pandemic income level. Thus, more research is needed to establish the precise relationships between mental health symptomatology and COVID-19. Finally, our sample was not completely representative of the United States population, limiting the generalizability of our results. Consequently, the effects of age, sex, and race on mental health outcomes in the US population overall merit further investigation.

Taken together, our findings provide important evidence demonstrating the factors contributing to human resilience as a crisis lingers. As such, these findings have real-world implications, serving as indicators of potential mental health vulnerabilities that could assist clinicians and policymakers as they allocate mental health resources during turbulent times. Such a tool would prove most imperative, given the long-lasting effects of the COVID-19 pandemic on both physical and mental health globally.

## Supplementary information

Supplemental Material
